# Effect of Dietary Selenium Yeast Supplementation on Porcine Circovirus Type 2 (PCV2) Infections in Mice

**DOI:** 10.1371/journal.pone.0115833

**Published:** 2015-02-27

**Authors:** Gang Liu, Guan Yang, Guiping Guan, Yuzhe Zhang, Wenkai Ren, Jie Yin, Yordan Martínez Aguilar, Wei Luo, Jun Fang, Xinglong Yu, Tiejun Li, Yulong Yin

**Affiliations:** 1 Scientific Observing and Experimental Station of Animal Nutrition and Feed Science in South-Central, Ministry of Agriculture, Hunan Provincial Engineering Research Center of Healthy Livestock and Poultry, Key Laboratory of Agro-ecological Processes in Subtropical Region, Institute of Subtropical Agriculture, Chinese Academy of Sciences, Changsha, Hunan, 410125, China; 2 College of Animal science & Technology, Hunan Agricultural University, 410128, Changsha, Hunan, China; 3 Graduate University of the Chinese Academy of Sciences, Beijing, 100039, China; 4 College of Veterinarian, Hunan Agricultural University, Changsha, Hunan, 410128, China; University of Liverpool, UNITED KINGDOM

## Abstract

The present study was performed to determine the protective role of dietary selenium (Se) yeast supplementation in porcine circovirus type 2 (PCV2) infected mice. Forty-eight Kun Ming female mice were randomly assigned to Se yeast group (0.3%Se +basal diet, n = 24) and control group (basal diet, n = 24). After 3 days of adaptive feeding and 15 days treatment with the experimental feed, mice were challenged by intraperitioneal injection of PCV2 at the dosage of 2000 TCID_50_ (50% tissue culture infection dose, TCID_50_). Serum total superoxide dismutase (SOD) activity, malondialdehyde (MDA) level, tumor necrosis factor alpha (TNF-α), C-reactive protein (CRP) and interleukin-1 beta (IL-1β) levels were measured at 5, 10, 15, 20 days post infection (dpi). The PCV2 virus load in the liver, spleen and lung, and the microscopic lesions in the liver, spleen and lung also were determined on 5, 10, 15, and 20 dpi. Dietary Se yeast supplementation decreased (Pμ0.05) the serum levels of TNF-α, but had no significant effect on the activity of SOD and the levels of MDA, CRP and IL-1β between experimental and control groups. Dietary Se yeast supplementation had little effect on the PCV2 virus load in the liver, spleen and lung. However, mice in the selenium yeast group showed a significant decrease in microscopic lesion scores in the lung and spleen compared with those in the control group (Pμ0.05). These data indicate Se yeast attenuated the PCV2 infection through altering the systemic inflammation and maintaining the normal organ morphology.

## Introduction

Porcine circovirus type 2 (PCV2) is highly pathogenic and is associated with a wasting disorder recognized as post-weaning multi-systemic wasting syndrome (PMWS) [1,2], porcine dermatitis and nephropathy syndrome (PDNS) [3], reproductive disorders [4], enteritis [5], proliferative and necrotizing pneumonia (PNP) [6] and porcine respiratory disease complex (PRDC) [7]. The virus is found in America, Europe, Australia and Asia, and therefore imposes a significant economic burden on the swine industry [8–12]. Nutritional regulation with immunoregulators to enhance the immune response might be useful as a prophylactic measure against PCV2 infection [13,14].

Selenium (Se) is an essential trace element in human nutrition. It is a functional component of the antioxidant defense system and is needed for the maintenance of the immune functions. In addition, it has also been reported that dietary Se supplementation can enhance the immune function [15,16], inhibit the activation of HIV-1 in cell culture through oxidative stress [17], and suppress the TNF-α-induced HIV replication in culture [18]. Moreover, Pan *et al* [19] reported that DL-selenomethionine inhibits the PCV2 replication *in vitro*. However, so far there is little data on the effects of selenium on PCV2 infection *in vivo*. Selenium yeast is a common form of selenium used to supplement the dietary intake [20]. Thus, in the present study, we evaluated the effects of dietary selenium yeast supplementation on the immunological responses against PCV2 in mice infected with PCV2. In this study, Mouse was used as the PCV2 infectious model because the previous investigations from others and our lab have shown that PCV2 could replicate in mouse model and cause microscopical lesion to organs similar to the observations in pigs [21–25]. In mouse model, PCV2 infection resulted in interstitial pneumonia and alveolar wall thickening in the lung, lymphohistiocytic inflammation in the liver, and lymphoid depletion and histiocytic inflammation in the spleen, which also been observed in PCV2 infected pigs [24,26,27].

## Materials and Methods

### Preparation of PCV2 stock

A PCV2 infectious clone constructed by self-ligation of the PCV2 genome via a *SacII*enzyme site was used to generate the virus stock pools used for experimental infection [13,14]. Briefly, the continuous porcine kidney cell line PK-15 [28], free of PCV1 and PCV2, was cultured in RPMI medium 1640 supplemented with 6% (vol/vol) fetal calf serum (FCS). The cell monolayer was dispersed by using trypsin-EDTA, and suspended in RPMI medium 1640 supplemented with 6% (vol/vol) FCS. Cells were simultaneously infected with the PCV2 infectious clone. After 72 h of incubation, the infected cells were frozen and thawed three times, and the cell mixture was tested by PCR before being stored at -20°C. PCV2 stocks were titrated on PK-15 cells [28].

### Animals and feeding

Forty eight KunMing mice (18–22 g) were obtained from the Animal Center of Central South University, Hunan, China. This strain of mice was originated from Swiss mice in the Indian Haffkine Institute in 1944 and showed high disease resistance, good adaptive capacity, high breeding coefficient and good survival rate. Now it is the most commonly used outbred mouse line in pharmacological, toxicological, medicinal and biological research and testing in China [29]. The mice were randomly assigned to two treatment groups: Se yeast group (0.3%Se yeast +basal diet, n = 24) and control group (basal diet, n = 24). The detail composition of basal diet was published in our previous study [30]. All mice received their respective dietary treatment throughout the experimental period. The Se yeast was generously provided by Hunan Agricultural University, Hunan, China. The Se yeast had a guaranteed analysis of 1g/kg of organically bound Se, with 78% being selenomethionine. The mice were housed in an environmentally controlled pathogen-free colony. This study was carried out in accordance with the Chinese guidelines for animal welfare and was approved by the Animal Care and Use Committee of the Chinese Academy of Sciences [13,14].

### Mouse infections

After 3 days of adaptive feeding and 15 days treatment with the experimental feed, mice were challenged by intraperitioneal injection of PCV2 at the dose of 2000 TCID_50_ (50% tissue culture infection dose, TCID_50_). All inoculated mice ate normally and did not appear unwell.

### Sample collection

On the day of necropsy, mice were anesthetized intraperitoneally with tribromoethanol (250 mg/kg body weight), and then killed by cervical dislocation. Six mice per treatment is sufficient to discern treatment effects based on our previously studies in mice [21,22,31–33]. Thus six mice per treatment were randomly killed at 5, 10, 15 and 20 dpi to collect blood and tissue samples. Serum was obtained by centrifugation at 2000g for 10 min and then stored at -80°C for further analysis. Liver, lung and spleen samples were collected and divided into two parts. One part stored at -80°C for DNA extraction and the other fixed in 10% formaldehyde for histopathologic and electron-microscopic examination.

### Serum and tissue analysis

Based on the description from Beauchamp and Fridovich [34], total serum superoxide dismutase (SOD) activity was measured at 525 nm using spectrophotometric kits (Nanjing Jiancheng Biotechnology Institute, China). MDA levelwas measured using spectrophotometric kits(Nanjing Jiancheng Biotechnology Institute, China), as described by Uchiyama and Mihara [35]. Serum tumor necrosis factor alpha (TNF-α), C-reactive protein (CRP) and interleukin-1 beta (IL-1β) levels were measured using enzyme linked immunosorbent assay (ELISA) (Cusbio biotech CO.,LTD, China) according to the manufacturer’s instructions.

### Histopathological and electron-microscopic examination

Samples of spleen, liver and lung from mice that exhibited macroscopic lesions were fixed in 10% neutral buffered formalin, embedded in paraffin, sectioned (5μm thickness), and stained with hematoxylin and eosin (H&E) for histopathological examination[14]. Microscopic lesions were evaluated in a blinded fashion by a veterinary pathologist using a previously described scoring system [26,27,36]. Lung sections were examined for the presence and severity of interstitial pneumonia, and scored on a scale from 0 (normal) to 6 (severe diffuse). Sections of liver were evaluated for the presence of lymphohistiocytic inflammation and scored on a scale from 0 (none) to 3 (severe). The spleen was evaluated for the presence of lymphoid depletion and histiocytic inflammation, and scored on a scale from 0 (normal) to 3 (severe).

### DNA extraction and PCV2 Real-time Quantitative PCR (qPCR) detection

DNA was extracted from samples [liver(10mg), spleen(5mg), lung (10mg)] using Tissue Genomic DNA Extraction Kits (Betimes Biotechnology Co., Ltd, China) according to the manufacturer’s instruction, eluted in 80 μl of elution buffer and stored at-20℃ until used for quantification of PCV2 genes by real time PCR. Briefly, a PCV2 genome was cloned in the pMD 18-T Vector (TaKaRa) after PCR amplification with following primers: forward, 5’- CCGCGGGCTGGCTGAACTTTTGAAAG-3’ and reverse, 5’- CCGCGGAAATTTCTGACAAACGTTAC -3’ (Genebank accession number: EU095020), and was transformed in TOP10 competent cells (Invitrogen). The plasmid was prepared using a PureLinkTM HiPure Plasmid Midiprep Kit (Invitrogen). The PCV2 plasmid was mixed with mouse DNA extracted from a PCV2 PCR-negative blood sample. Ten-fold dilutions of this mixture (from 10^11^ to 10^2^ PCV2 copy numbers/ul) were used as standard for PCV2 quantitation of samples. PCR was performed using SYBR Green detection kit (Takara, China), containing MgCl_2_, dNTP, and Hotstar Taq polymerase. One μl of template solution was added to a total volume of 10 μL containing 5μL SYBR Green mix, and 0.2 μL each of the forward and reverse primers (10uM). We used the following protocol: (i) pre-denaturation (30 s at 95°C); (ii) amplification and quantification, repeated 40 cycles (5 s at 95°C, 34 s at 60°C); and (iii) melting (60–99°C at a heating rate of 0.1°C/s and fluorescence measurement)[14].

### Statistical analysis

All statistical analyses were performed using the SPSS 17.0 software (Chicago, IL, USA). Group comparisons between controls and selenium group at each time points were performed using Student’s t-test. Data are expressed as mean ±standard error of the mean (SEM). P < 0.05 was considered statistically significant.

## Results

### Antioxidant enzymes and cytokines

The serum SOD activity and MDA content are shown in Table 1. No differences in the SOD activity and MDA level were detected between the two groups (P >0.1). The serum level of TNF-α in the selenium yeast group was significantly lower than the control group (Table 2) at 5 (P = 0.004), 10 (P = 0.019), 15 (P = 0.045), and 20 (P = 0.013) dpi respectively. However, no significant difference between the selenium and the control groups was observed for CRP (Table 2) and IL-1β (Table 2).

**Table 1:** Effects of dietary selenium yeast supplementation on serum superoxide dismutase (SOD) activity and malondialdehyde (MDA) level.

Items	5dpi	10dpi	15dpi	20dpi
SOD(U/ml)				
Selenium group	213.29±9.63	205.92±8.19	226.72±10.86	205.40±6.23
Control group	211.52±8.65	196.29±11.63	222.44±8.79	209.36±15.29
MDA(nmol/ml)				
Selenium group	13.10±3.15	13.72±3.86	13.86±3.66	16.71±6.78
Control group	15.43±3.16	14.82±4.11	10.54±1.56	19.15±2.65

Data are mean ± SEM, n = 6. SOD: superoxide dismutase, MDA: Malondialdehyde, Mice in Selenium yeast group are fed with 0.3%Se yeast +basal diet; while mice in control group are fed with basal diet.

**Table 2:** Effects of dietary selenium yeast supplementation on serum levels of C-reactive protein (CRP), tumor necrosis factor alpha (TNF-α) and interleukin-1 beta (IL-1β).

Items	5dpi	10dpi	15dpi	20dpi
CRP(μg/ml)				
Selenium group	92.10±14.32	73.09±4.85	83.22±7.02	77.81±9.33
Control group	76.89±3.74	90.27±8.73	88.71±2.18	63.10±19.23
TNF-α(pg/ml)				
Selenium group	43.00±1.66**	81.83±19.54*	82.38±18.79*	65.73±28.98*
Control group	130.14±18.76	420.02±97.67	168.03±28.23	374.45±65.67
IL-1β(ng/ml)				
Selenium group	35.16±1.77	29.11±1.99	33.80±2.21	28.37±2.32
Control group	32.24±1.65	29.96±2.51	29.47±2.87	27.78±2.10

Data are mean ± SEM, n = 6. Selenium group means 0.3%selenium yeast +basal diet, control group means basal diet. CRP: C-reactive protein, TNF-α: Tumor necrosis factor alpha, IL-1β: Interleukin-1 beta.

**Mean values sharing different superscripts within a column differ (P< 0.01).

* Mean values sharing different superscripts within a column differ (P< 0.05).

### PCV2 virus load in liver, spleen and lung

At 5 dpi, PCV2 DNA was detected in 8/10 liver samples, 5/10 spleen samples and 10/10 lung samples in the selenium group, and the mean PCV2 log10 genomic copies per gram were 3.9±0.2, 5.7±1.0, 4.3±0.1 for the liver, spleen and lung, respectively. In the control, PCV2 DNA was detected in 8/10 liver samples and 8/10 lung samples and the mean PCV2 log10 genomic copies per gram were 6.4±1.3, 9.2±1.6 for the liver and lung, respectively (Table 3). At 10 dpi, PCV2 DNA was only detected in selenium group in 8/10 of the lung, the mean PCV2 log10 genomic copies per gram were 4.3±0.2 (Table 3). At 15 dpi, PCV2 DNA was detected in 10/10 liver samples, 4/10 spleen samples and 5/10 lung samples in selenium group, the mean PCV2 log10 genomic copies per gram were 4.5±0.3, 11.3±1.5, 4.7±0.9 for the liver, spleen and lung, respectively. While in control group were 8/10 liver samples, 4/10 spleen samples and 5/10 lung samples, with the mean PCV2 log10 genomic copies per gram of 5.5±1.0, 12.6±0.8, and 5.0±0.6 for the liver, spleen and lung, respectively (Table 3). At 20 dpi, PCV2 DNA was detected in 7/10 liver samples and 4/10 spleen samples in selenium group and the mean PCV2 log10 genomic copies per gram were 4.1±0.3 and 8.9±2.0 for the liver and spleen, respectively. In control group was 8/10 lung samples and the mean PCV2 log10 genomic copies per gram was 7.1±1.4 (Table 3).

**Table 3:** PCV2 virus load in the liver, spleen and lung in selenium yeast group and control group.

Items	5dpi	10dpi	15dpi	20dpi
Liver				
Selenium group	8/10(3.9±0.2)	0/10(0.0±0.0)	10/10(4.5±0.3)	7/10(4.1±0.3)**
Control group	8/10(6.4±1.3)**	0/10(0.0±0.0)	8/10(5.5±1.0)	0/10(0.0±0.0)
Spleen				
Selenium group	5/10(5.7±1.0)**	0/10(0.0±0.0)	4/10(11.3±1.5)	4/10(8.9±2.0)**
Control group	0/10(0.0±0.0)	0/10(0.0±0.0)	4/10(12.6±0.8)	0/10(0.0±0.0)
Lung				
Selenium group	10/10(4.3±0.1)	8/10(4.3±0.2)**	5/10(4.7±0.9)	0/10(0.0±0.0)
Control group	8/10(9.2±1.6)**	0/10(0.0±0.0)	5/10(5.0±0.6)	8/10(7.1±1.4)**

No. positive mice /No. tested mice, PCV2: porcine circovirus type 2, the data are shown as the mean PCV2 log10 genomic copies per gram samples ±standard error of the mean (SEM). Mice in Selenium yeast group are fed with 0.3%Se yeast +basal diet; while mice in control group are fed with basal diet.

**Mean values sharing different superscripts within a column differ (P< 0.01).

### Pathological examination

**Fig 1:**
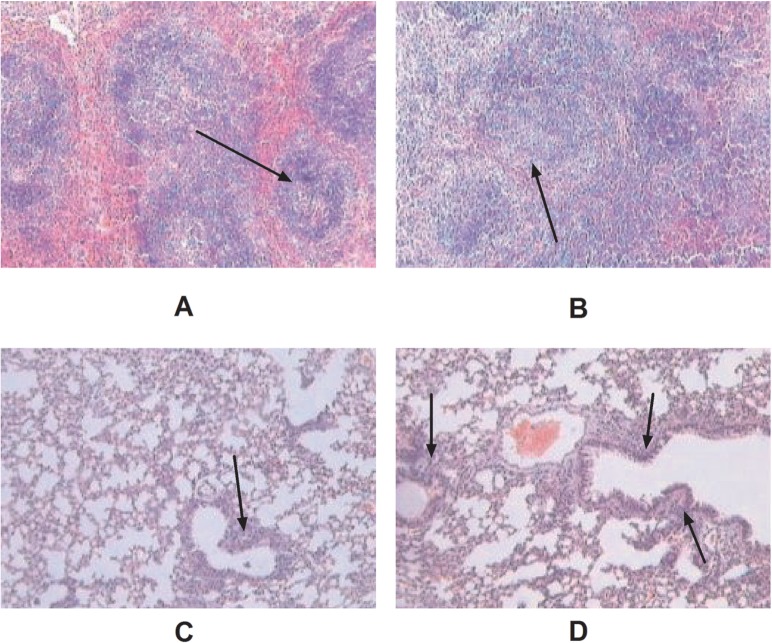
Histopathological findings in the lung and spleen(×100). The selenium yeast group shows less-severe lymphoid depletion (A) in the spleen compared with the control group (B). Additionally, mild interstitial pneumonia is evident in the lung in the selenium yeast group (C) compared with the control group (D). Mice in Selenium yeast group are fed with 0.3%Se yeast +basal diet; while mice in control group are fed with basal diet.

Microscopic lesions in the spleen were observed from 5 to 20 dpi in both groups; and dietary supplementation with Se yeast significantly decreased the microscopic lesions in the spleen (Fig. 1A vs. Fig. 1B). Unlike the spleen, microscopic lesions in lung were observed from 10 dpi in control group, but only at 20 dpi in Se yeast group. However, the lesions in the lung in the Se yeast group (Fig. 1C) were not as severe as those in the control group (Fig. 1D). Microscopic lesions were found in the liver in both groups in the experimental period (Table 4). Mice in the Se yeast group showed a significant decrease in microscopic lesion scores in the lung and spleen compared with those in the control group (P<0.05).

**Table 4:** Microscopic lesion scores for tissue samples in PCV2-infected mice.

Items	5dpi	10dpi	15dpi	20dpi
Lung				
Selenium group	0	0	0	1.00±0.57
Control group	0	1.33±0.32**	0.67±0.34	2.34±0.87*
Spleen				
Selenium group	1.25±0.25	0.75±0.25	1.40±0.24	0.60±0.24
Control group	1.80±0.37*	1.00±0.40	1.50±0.27	1.40±0.50**
Liver				
Selenium group	0	0	0	0
Control group	0	0	0	0

PCV2: porcine circovirus type 2. Mice in Selenium yeast group are fed with 0.3%Se yeast +basal diet; while mice in control group are fed with basal diet.

**Mean values sharing different superscripts within a column differ (P< 0.01).

* Mean values sharing different superscripts within a column differ (P< 0.05).

## Discussion

A recent study showed that Se may be used to inhibit PCV2 replication[19]. PCV2 is recognized as the essential infectious agent of many several serious porcine diseases such as PMWS and PRDC [37,38] that cause large economic losses worldwide. However, there is paucity of data on the effects of dietary Se supplementation on PCV2 infection. Therefore, we evaluated the effects of Se yeast on the control of PCV2 replication.

We measured the SOD activity and the MDA content, because antioxidant enzymes play major roles in the protection of biological macromolecules against peroxidative damage [39]. SOD accelerates the dismutation of superoxide (O_2_^2-^) into hydrogen peroxide (H_2_O_2_), and represents the step in the prevention of the generation of free radicals under physiological conditions. MDA, a major oxidation product of peroxidized polyunsaturated fatty acids, is another indicator for oxidative stress[40]. We observed no statistically differences of the SOD activity between the Se yeast group and control group, in apparent disagreement with previous studies, showing that dietary Se supplementation increases the SOD activity and reduced MDA level [41–43].

Cytokines are a large family of proteins that play important roles in innate and adaptive immune systems. CRP plays a role in host defense against bacterial pathogens [44,45]. TNF-α has a key role in immune regulation, increasing lymphoid development, cell proliferation, differentiation, activation and death [46,47]. IL-1β is a pre-inflammatory cytokine, which is secreted by polymorphonuclear leukocyte and monocytes [48]. It enables organisms to respond to infectious non-self challenges and induces a cascade of effects leading to inflammation through up- or down-regulation of other cytokines [49]. In our study, the serum level of TNF-α in the Se yeast group is significantly lower than the controls. Likewise, previous study also found that Se supplementation lowers the production of TNF-α in other diseases or infectious models [50,51]. Indeed, TNF-α production in mice alveolar macrophages, which experimentally infected with PCV2, is reported to be higher than the control group [52]. Also in PCV2-inoculated alveolar macrophages, the level of TNF-α is significantly increased [53]. These results are indicating that selenium supplementation maybe be beneficial in PCV2 infected mice. However, no statistically differences between the experimental group and the control group are observed for CRP and IL-1β.

An important part of this study is the comparison of the microscopic lesions in the tissue from the Se yeast group and control group. As noticed in previous studies, the PCV2-infection is associated with lymphoid depletion and histiocytic inflammation of the spleen [14]. The lesions in lung are characterized by interstitial pneumonia and alveolar wall thickening due to macrophages and lymphocytes [14]. Previous reports and observations showed that PCV2-infection is associated with lymphohistiocytic inflammation in the liver [14]. In the present study, we found that dietary Se yeast supplementation appears to have a positive effect on PCV2 induced lymphoid depletion and histiocytic inflammation. In this study, we also detected the PCV2 virus load in the tissue to validate the beneficial role of Se supplementation, however, the virus is sporadically detected in such a way that it could not be statistically analyzed at every time point. The reasons for this irregular detection are various, and we have stated it in our previous papers [21,25,26].

Several reasons may be ascribed to the lack of a more profound effect in the present study. The most possible reason may be the timing of PCV2 injection. In many other positive cases, the researchers fed the prepared feed to the mice at least for 3–4 consecutive weeks before initial treatment [54,55]. Some researchers have also suggested that the earlier PCV2 infection occurs, the higher the risk of pigs developing PMWS [56,57]. Since we fed the prepared feed for 15 days before injection, the Se yeast might not have been long enough to have the necessary effect. The consecutive feeding time of Se yeast on the PCV2 therefore needs further research. Another important reason that is that our study was conducted under controlled, animal-friendly environment. Under field conditions, porcine parvovirus [58], porcine reproductive and respiratory syndrome virus (PRRSV) [59] and mycoplasma hyponeumoniae [27] all may enhance PCV2-associated lesions and increase the incidence of PMWS [60,61]. Since PCV2 can be present in healthy pigs [37], we hypothesized that, in an animal- friendly environment, the PCV2 infection may be attenuated by the good sanitary conditions in the absence of additional ‘trigger’ factors.
